# Effect of Introducing Xpert MTB/RIF to Test and Treat Individuals at Risk of Multidrug-Resistant Tuberculosis in Kazakhstan: A Prospective Cohort Study

**DOI:** 10.1371/journal.pone.0132514

**Published:** 2015-07-16

**Authors:** Sanne Christine van Kampen, Aigul Tursynbayeva, Aliya Koptleuova, Zauresh Murzabekova, Lyazzat Bigalieva, Moldir Aubakirova, Svetlana Pak, Susan van den Hof

**Affiliations:** 1 KNCV Tuberculosis Foundation, Central Office, The Hague, Netherlands; 2 KNCV Tuberculosis Foundation, Central Asia Office, Almaty, Kazakhstan; 3 National Center of Tuberculosis Control, Almaty, Kazakhstan; 4 Akmola Regional Tuberculosis Dispensary, Kokshetau, Kazakhstan; 5 East Kazakhstan Regional Tuberculosis Dispensary, Oskemen, Kazakhstan; 6 Almaty City Tuberculosis Dispensary, Almaty, Kazakhstan; McGill University, CANADA

## Abstract

**Background:**

Xpert MTB/RIF (Xpert) was piloted in Kazakhstan to detect tuberculosis (TB) and rifampicin resistance (RR-)TB among individuals at risk of multidrug-resistant (MDR-) TB. This study assessed the performance of Xpert compared to conventional diagnostic methods, RR-TB case detection among various risk groups, treatment initiation and time to diagnosis and treatment.

**Methods:**

Eligible individuals were tested with Xpert, smear microscopy, culture and drug-susceptibility testing (DST) at the national TB reference laboratory and three provincial laboratories. Data was collected prospectively from August 2012 to May 2013 from routine laboratory and treatment registers.

**Results:**

A total of 5,611 Xpert tests were performed mostly targeting contacts of MDR-TB patients, ‘other’ presumptive MDR-TB patients, and retreatment cases (26%, 24% and 22%, respectively). Compared to phenotypic DST, the positive predictive value of Xpert to detect RR-TB was 93.1% and 96.4% and the negative predictive value was 94.6% and 92.7% using solid and liquid culture media, respectively. RR-TB detection was highest among (former) prisoners, retreatment cases, people living with HIV/AIDS (PLWHA), and TB patients with positive smears after intensive phase of treatment (59%, 58%, 54% and 53% among TB positives, respectively). 88.9% of RR-TB patients were registered to have started second-line TB treatment. Median time to diagnosis with Xpert was 0.0 days (IQR 0.0-1.0), time from diagnosis to start of first-line treatment 3.0 days (IQR 1.0-7.0), and to start of second-line treatment 7.0 days (IQR 4.0-16).

**Conclusions:**

Compared to conventional culture and DST, Xpert had a shorter result turn-around-time and excellent concordance to detect RR-TB. Time from sputum collection to start of second-line treatment was reduced to one week. The yield of Xpert could be maximized by increasing referrals from penitentiary and HIV centers to TB centers.

## Introduction

Kazakhstan is among the 27 high multidrug-resistant tuberculosis (MDR-TB) burden countries in the world with an estimated 23% of MDR-TB among new TB patients and 55% among retreatment TB patients.[1] In 2012, the National TB Control Program (NTP) detected 7,608 out of 8,800 (86%) of estimated MDR-TB cases, owing to availability of culture and drug-susceptibility testing (DST) in all provincial laboratories (6.8 laboratories per 5 million population).[1] Conventional diagnostic practice in Kazakhstan is to test all individuals at risk of TB with sputum smear microscopy and culture. Positive culture isolates are investigated further for first-line DST and second-line DST in case of first-line drug-resistance. Out of 7,608 MDR-TB cases notified in 2012, 7,213 (94.8%) were started on second-line treatment, and treatment success in the 2009 second-line treatment cohort was 73%, which is high compared to other countries.[1–3] Practice is to hospitalize all TB and MDR-TB patients during the intensive phase of treatment, as well as smear-negative TB patients, although efforts are ongoing to move towards more outpatient care.[2, 4] Finally, Kazakhstan has a low burden of TB/HIV co-infection, with only 2.1% of TB patients being HIV positive in 2012.[1] However, the most common cause of death among people living with HIV/AIDS (PLWHA) is TB disease.[5]

Although case detection and treatment initiation for MDR-TB are high in Kazakhstan, time to diagnosis and subsequent treatment is still long due to the time needed to obtain culture and DST results. A new rapid molecular test to detect TB and rifampicin resistant (RR-)TB, Xpert MTB/RIF assay (Xpert) has been endorsed by the World Health Organization (WHO) in December 2010.[6] When used as initial test replacing smear microscopy, Xpert detects TB with a median sensitivity of 89% and specificity of 99% compared to culture. When it replaces phenotypic DST, Xpert detects RR-TB with a median sensitivity of 95% and specificity of 98%.[7] Moreover, while culture and DST requires several weeks to months to produce a result, an Xpert result is available within two hours. RR-TB is a good proxy of MDR-TB, since in most settings resistance to rifampicin is regularly associated with resistance to isoniazid, particularly where fixed-dose combination first-line anti-TB drugs are used.[8]

Following WHO recommendations, in 2012 the NTP of Kazakhstan decided to implement Xpert with the aim to improve case finding and management of MDR-TB patients by reducing time to diagnosis and allow earlier initiation of appropriate treatment. The procurement and installation of the first Xpert instruments in Kazakhstan was supported by USAID as part of a larger global health and development initiative. This article describes the outcomes of a pilot study in four sites to assess the performance of Xpert compared to culture and DST, TB and RR-TB case detection among various risk groups, treatment initiation and time to diagnosis and treatment. The outcomes were used to inform the NTP on the effects of Xpert implementation and provide input for scale-up strategies.

## Study Population and Methods

**Fig 1:**
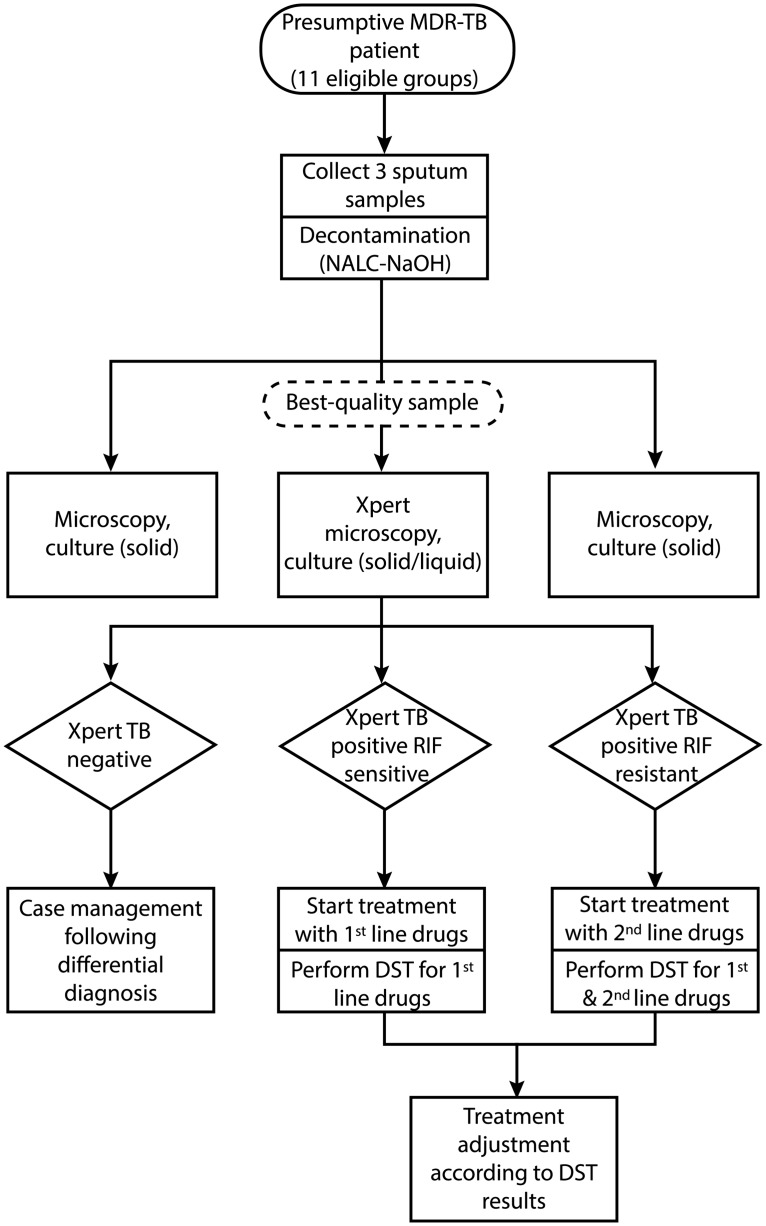
Diagnostic and treatment algorithm for presumptive multidrug-resistant TB cases registered at four sites in Kazakhstan from 1 August 2012 to 31 May 2013. Abbreviations: MDR-TB, multidrug-resistant tuberculosis; NALC-NaOH, N-acetyl L-cystein and sodium hydroxide; RIF, rifampicin; DST, drug-susceptibility testing.

This study included all individuals tested with Xpert from 1 August 2012 to 31 May 2013 (10 months) at four pilot sites: the National TB Reference Laboratory (NRL) (covering Talgar region and patients from the National TB Center), Almaty province laboratory, Akmola province laboratory, and East-Kazakhstan regional laboratory. In consultation with the NTP, eleven groups at risk of MDR-TB were eligible for Xpert testing as listed in Table 1. The diagnostic and treatment algorithm used for eligible individuals in this study is shown in Fig 1. Xpert testing was added to the conventional algorithm. In line with routine practice, three sputum samples per individual were collected and decontaminated. Smear microscopy, solid media culture (Löwensten-Jensen), liquid media culture (Bactec MGIT 960), and Xpert were performed in parallel on the sample with the highest volume and purulence. The other two samples received smear and solid media culture only. Positive culture isolates were inoculated for first-line DST.

**Table 1:** Eleven groups at risk of multidrug-resistant TB eligible for Xpert MTB/RIF testing in four pilot sites in Kazakhstan.

**Presumptive MDR-TB patients who have had previous TB treatment**:
1. Retreatment cases: treatment failure, relapse, treatment after default
2. TB patients receiving category I, II, III treatment with positive sputum smear result at the end of intensive phase in case of absence of DST results or in case of suspected development of drug resistance during treatment
3. Patients with TB/HIV co-infection without DST data
4. Patients who were previously treated with regimens which are not in accordance with Kazakhstan National TB guidelines
**Presumptive MDR-TB patients without previous TB treatment, but with symptoms of TB and/or abnormalities on chest X-ray**:
5. Close contacts of MDR-TB patients
6. Persons in prison or after their release, because of perceived risk of contracting MDR-TB in prison
7. Pregnant or postpartum women, because of perceived risk of rapid progression to serious disease
8. Patients with acute progressive forms of tuberculosis: caseous pneumonia, generalized forms of TB, including miliary tuberculosis
9. People living with HIV/AIDS, because of documented links between HIV infection and MDR-TB in the European region. [11]
10. Medical and prison staff, because of high rate of notified TB among health care workers of 204 per 100.000. [1]
11. Other (as identified by clinician)

Abbreviations: MDR-TB, multidrug-resistant tuberculosis; DST, drug-susceptibility testing.

Individual patient data was collected from electronic laboratory and treatment registers and copied to Excel: identification number, date of birth, sex, referring hospital, eligible group, test results of Xpert, smear microscopy, culture and DST, date of sputum collection, sputum referral, test initiation and result release, treatment category and date of treatment start. After registration was closed, data collection continued for another two months until 31 July 2013 to allow inclusion of outstanding test results and treatment information. Data was validated by checking at least 10% of records against the original registers at each site. There were no major issues with quality of data transfer, but the original registers were incompletely filled, especially in two of the sites. There, it was very difficult to obtain complete treatment information as most presumptive MDR-TB patients were referred from and returned for treatment to remote district TB facilities (Site 1), pre-trial detention centers, the penitentiary system, or HIV centers (Site 2). A decision was made to exclude treatment data from these two sites. Data cleaning was done by correcting for double entries, erroneous dates (e.g. date of testing before date of release of test result) and erroneous results (e.g. Xpert TB negative rifampicin positive result) in consultation with staff on-site. Data analysis was done in IBM SPSS 21. Performance of Xpert was assessed by comparing test results of Xpert and conventional phenotypic DST within the same individuals and calculating concordance, negative and positive predictive values (NPV and PPV), proportion of unsuccessful tests and time to diagnosis. Time to diagnosis was taken as the number of days from sputum collection to release of Xpert or culture result, respectively (date of DST result was not recorded). Only in Site 1, date of sputum collection for culture was not recorded and date of culture inoculation was used instead. Xpert case detection was analyzed by calculating proportions of individuals tested and detected with TB or RR-TB for each of the eleven risk groups. Appropriate treatment initiation was assessed by calculating proportions of diagnosed patients with registered first- and second-line TB treatment, and the number of days to start treatment for each of the four pilot sites.

The National Center for TB Control in Almaty, Kazakhstan, authorized the study protocol based on adherence to an appropriate ethical framework for using patient data, which included the collection of data solely from routine national databases and de-identification and anonymous analysis of patient data.

## Results

### Performance of Xpert compared to conventional diagnosis

In total, 5,611 individuals were tested with Xpert in four sites, with an average monthly number of tests of 159 per site during the last three months of the study. Overall, 500 individuals (8.9%) had an unsuccessful Xpert test (i.e. error, invalid or no result); this varied from 5.1% in Site 1 to 16.9% in Site 3, where power fluctuations led to a high frequency of aborted tests. The median time to diagnosis with Xpert was 1.0 day or less (interquartile range (IQR) 0.0–1.0) in all sites.

Among 5,611 individuals, 4,259 (75.9%) had a registered result for smear microscopy, 1,819 (32.4%) for solid media culture and 2,750 (49.0%) for liquid media culture. Smear microscopy detected 1,234 TB positive cases (59 of them were negative with Xpert), while Xpert detected an additional 496 (28.7%). Proportion of unsuccessful (i.e. contaminated) culture tests was 1.9% with solid media and 4.9% with liquid media. Time to diagnosis was a median of 48 days (IQR 42–73) with solid media and 36 days (IQR 11–43) with liquid media. Thus compared to conventional culture, Xpert had 47 days and 35 days shorter turn-around-time and 7.0% and 4.0% higher proportion of unsuccessful test results than solid and liquid media culture, respectively.

Among culture TB positives, 70.7% (428/605) had a DST result with solid media recorded and 81.9% (871/1063) with liquid media. When Xpert rifampicin results were compared to conventional DST, 94.6% and 92.7% of individuals with an Xpert rifampicin susceptible result also tested susceptible with DST using solid and liquid media, respectively (NPV) (Table 2). The proportion of Xpert rifampicin resistant individuals that had a resistant DST result was 93.1% and 96.4% with solid and liquid media, respectively (PPV). The concordance between Xpert and phenotypic DST to detect RR-TB was 93.1% (351/377) with solid media and 93.9% (636/677) with liquid media. Out of 59 individuals with discrepant Xpert and DST results for rifampicin resistance, 33 (55.9%) had treatment data recorded and among them, 27 (81.8%) subsequently started TB treatment in line with the Xpert result, while 6 (18.2%) started in line with the DST result.

**Table 2:** Detection of rifampicin resistant TB using Xpert MTB/RIF, solid and liquid media DST within the same individuals at four provincial laboratories.

	Results of solid media DST			Results of liquid media DST		
Results of Xpert	RIF susceptible *n* (%)	RIF resistant *n* (%)	Total *n*	RIF susceptible *n* (%)	RIF resistant *n* (%)	Total *n*
RIF susceptible	176 (94.6)	10 (5.4)	186	292 (92.7)	23 (7.3)	315
RIF resistant	13 (6.9)	175 (93.1)	188	13 (3.6)	344 (96.4)	357
RIF indeterminate	2 (66.7)	1 (33.3)	3	3 (60.0)	2 (40.0)	5
Total	191	186	377	308	369	677

Abbreviations: RIF, rifampicin; DST, drug-susceptibility testing.

### Case detection with Xpert among various risk groups

Overall, most individuals tested with Xpert were close contacts of MDR-TB patients (25.5%), ‘other’ presumptive MDR-TB patients (23.6%), and retreatment cases (22.2%) (Table 3). For the group of ‘Others’, not all clinicians provided more detailed information on reason for testing. This group was subdivided into presumptive TB patients (32.0%), newly diagnosed TB patients (25.0%) and unspecified others (43.0%). The distribution of risk groups varied per site. More than half of individuals registered as ‘Others’ or with missing information on risk group were registered at Site 1.

**Table 3:** Number of individuals tested with Xpert MTB/RIF at four provincial laboratories from 1 Augustus 2012 to 31 May 2013.

	TOTAL *n* (%)	Site 1 *n* (%)	Site 2 *n* (%)	Site 3 *n* (%)	Site 4 *n* (%)
**Presumptive MDR-TB patients treated previously for TB**					
Retreatment patients	1,246 (22.2)	135 (8.0)	291 (20.7)	437 (37.4)	383 (28.9)
Smear-positive after intensive phase	357 (6.4)	57 (3.4)	47 (3.3)	82 (7.0)	171 (12.9)
TB/HIV co-infected patients	52 (0.9)	3 (0.2)	17 (1.2)	10 (0.9)	22 (1.7)
Previous treatment not in line with national guidelines	22 (0.4)	3 (0.2)	4 (0.3)	4 (0.3)	11 (0.8)
**Subtotal**	**1,814 (32.3)**	**221 (13.2)**	**377 (26.9)**	**547 (46.8)**	**671 (50.7)**
**Presumptive MDR-TB patients not previously treated for TB**					
Close MDR-TB contacts	1,432 (25.5)	350 (20.9)	441 (31.4)	318 (27.2)	323 (24.4)
Others: Not specified	571 (10.2)	421 (7.5)	50 (0.9)	1 (0.0)	99 (1.8)
Others: Presumptive TB cases	424 (7.6)	260 (4.6)	97 (1.7)	63 (1.1)	4 (0.0)
Others: Newly diagnosed TB cases	332 (5.9)	96 (1.7)	85 (1.5)	146 (2.6)	5 (0.0)
(Former)prisoners suspected of TB	205 (3.7)	7 (0.4)	84 (6.0)	65 (5.6)	49 (3.7)
Pregnant women or after delivery suspected of TB	199 (3.5)	13 (0.8)	153 (10.9)	7 (0.6)	26 (2.0)
Acute progressive TB	137 (2.4)	23 (1.4)	18 (1.3)	14 (1.2)	84 (6.3)
PLWHA suspected of TB	126 (2.2)	15 (0.9)	98 (7.0)	1 (0.0)	12 (0.9)
Medical/prison staff suspected of TB	62 (1.1)	32 (1.9)	5 (0.4)	13 (1.1)	12 (0.9)
**Subtotal**	**3,351 (59.7)**	**1,194 (71.2)**	**1,013 (72.2)**	**614 (52.5)**	**530 (40.1)**
Group type not recorded	446 (7.9)	263 (15.7)	53 (3.8)	8 (0.7)	122 (9.2)
**Total**	**5,611 (100)**	**1,678 (100)**	**1,442 (100)**	**1,169 (100)**	**1,322 (100)**

Abbreviations: MDR-TB, multidrug-resistant TB; PLWHA, people living with HIV/AIDS.

Among presumptive MDR-TB patients previously treated for TB, 57.0% tested TB positive with Xpert and among them, 54.9% had a RR-TB result (Table 4). Among presumptive MDR-TB patients that had not been treated for TB before, 35.5% was positive for TB with Xpert and among them 42.3% had a RR-TB result. (Former) prisoners as well as PLWHA suspected of TB had a high proportion of RR-TB similar to that of retreatment patients and patients with no sputum conversion after intensive phase of TB treatment, but they were only tested in small numbers.

**Table 4:** Proportion of TB positive and rifampcin resistant results among individuals successfully tested with Xpert MTB/RIF at four provincial laboratories.

Eligible group	Successfully tested *n* ^a^	TB positive *n*	Proportion TB positives among all tested %	Rifampicin resistant *n*	Proportion rifampicin resistant among TB positives %
**Presumptive MDR-TB patients treated previously for TB**					
Retreatment patients	1,114	638	57.3	368	57.8
Smear-positive after intensive phase	338	198	58.8	105	53.0
TB/HIV co-infected patients	48	31	64.6	13	41.9
Previous treatment not in line with national guidelines	21	9	42.9	3	33.3
**Subtotal**	**1,648**	**940**	**57.0**	**516**	**54.9**
**Presumptive MDR-TB patients not previously treated for TB**					
Close MDR-TB contacts	1,288	505	39.2	219	43.4
Oth ers: Not specified	571	157	27.5	69	43.9
Others: Presumptive TB cases	424	114	26.9	53	46.5
Others: Newly diagnosed TB cases	332	166	50.0	44	26.5
(Former)prisoners suspected of TB	188	68	36.2	40	58.8
Pregnant women or after delivery suspected of TB	190	25	13.2	8	32.0
Acute progressive TB	127	64	50.4	27	42.2
PLWHA suspected of TB	110	37	33.6	20	54.0
Medical/prison staff suspected of TB	56	14	25.0	6	42.9
**Subtotal**	**3,056**	**1,086**	**35.5**	**459**	**42.3**
Group type not recorded	407	168	41.3	73	43.5
**Total**	**5,111**	**2,194**	**42.9**	**1,048**	**47.8**

Abbreviations: MDR-TB, multidrug-resistant TB; PLWHA, people living with HIV/AIDS.

^a^ Excluding 500 unsuccessful Xpert tests with an error, invalid or no result.

### Appropriate treatment initiation after diagnosis with Xpert

In Sites 3 and 4, 88.9% of Xpert-detected RR-TB individuals were registered to have started second-line treatment, 94.8% of them had dates recorded, and among them the median time from Xpert result release to start of second-line treatment was 7.0 days (IQR 4.0–16) (Table 5). Notably, 6.8% had started second-line treatment before being tested with Xpert and culture. Among Xpert rifampicin susceptible individuals, 58.3% had started first-line treatment recently (median of 11 days, IQR 4.0–35) before being tested with Xpert. A small number had started second-line treatment either before (1.4%) or after (2.2%) testing. Of the remaining 38.1%, 94.3% (182/193) of patients newly started first-line treatment, 98.4% of them had dates recorded, and among them the median time from result release to start of first-line treatment was 3.0 days (IQR 1.0–7.0).

**Table 5:** Number and proportion of individuals tested with Xpert MTB/RIF that started first- or second-line TB treatment, with test and treatment date, and time from test result to start of treatment at two provincial laboratories.

Results of Xpert	Individuals starting treatment						With date of treatment *n* (%)	Median time to start treatment days (IQR)
	Total *n*	FLD before testing *n* (%)	FLD after testing *n* (%)	SLD before testing *n* (%)	SLD after testing *n* (%)	Missing data *n* (%)		
TB positive rifampicin resistant	559	13 (2.3)	0 (0.0)	38 (6.8)	497 (88.9)	11 (2.0)	471 (94.8) (471/497)	7.0 (4.0–16)
TB positive rifampicin susceptible	506	295 (58.3)	182 (36.0)	7 (1.4)	11 (2.2)	11 (2.2)	179 (98.4) (179/182)	3.0 (1.0–7.0)

Abbreviations: FLD, fist-line anti-TB drugs; SLD, second-line anti-TB drugs; IQR, interquartile range.

## Discussion

The main aim of introducing Xpert in Kazakhstan was to reduce time to diagnosis and allow earlier initiation of appropriate treatment of MDR-TB patients. Most presumptive (RR-) TB patients in Kazakhstan are hospitalized and initiated on first-line TB drug regimens while waiting for culture and DST results.[2] Indeed this study showed that almost 60% of Xpert rifampicin susceptible individuals had started first-line treatment before being tested. The few Xpert TB positive individuals who started second-line TB treatment before release of test results could have been treated empirically or have previous culture and DST results recorded elsewhere. After Xpert introduction, the average median time to start second-line treatment was shortened to one week, while previously it took 1–1.5 months to merely obtain a liquid and solid media culture result, respectively. Second-line TB treatment initiation of 89% in Sites 3 and 4 after detection with Xpert was high compared to findings from studies in Indonesia and South Africa.[9, 10] First- and second-line drugs were available continuously and free of charge, funded partly by the government and partly by Global Fund.[11] However, this proportion was somewhat lower than the national coverage rate of 95% as reported by WHO, which may indicate that treatment registration was incomplete. This problem could be solved by re-training clinicians and statistical staff.

Reduced time to diagnosis helps the patient to start appropriate treatment sooner, which will reduce the chance of developing or amplifying drug-resistance and could positively affect treatment outcomes. Besides, it could reduce disease transmission in the community. Combined with recent progress in Kazakhstan to introduce outpatient care for MDR-TB patients,[12] the introduction of Xpert could improve adequate treatment of MDR-TB and reduce the risk of nosocomial MDR-TB infection.

Concordance between Xpert and solid and liquid media culture DST to detect RR-TB was above 90%. The few patients with discrepant results were principally treated based on their Xpert result. This study did not follow up the patients after treatment initiation. Further studies should assess treatment response for patients with discrepant Xpert and DST results. Also, qualitative studies could investigate why physicians did not change treatment regimen after DST results were released. Compared to conventional culture, Xpert had a 4–7% higher proportion of unsuccessful test results in all sites. While in other countries, error rates were reduced by revision of procedures or module replacements,[13] in Kazakhstan the installation of surge protectors together with Xpert instruments and an uninterrupted power supply system drastically decreased the number of aborted tests to 6.7% in Site 3 and 2–3% in the other sites by the end of 2013. Since there was no protocol on repeating unsuccessful Xpert tests, clinicians based treatment decisions for these individuals on conventional diagnosis.

This study had several limitations. First of all, although all individuals should have received an Xpert and culture test, more than half of individuals had no culture result recorded and more than one-sixth of culture TB positive individuals had a missing DST result. The occurrence of missing culture and DST results was independent of whether Xpert was RIF susceptible or resistant, thus the risk of partial verification bias when comparing both tests was negligible. Staff on-site confirmed that missing results were due to flaws in recordings, not due to lack of testing. An earlier study evaluating completeness of DST results among culture-positive TB patients in Kazakhstan found that the rate of missing records decreased from 32.3% to 7.2% from March 2009 to March 2010 after regional TB centers were provided with monthly notifications to complete and correct data.[14] Our findings suggest that the completeness of records has reduced again, due to the introduction of a revised TB register, which does not have checks in place yet.

A second limitation was that the exclusion of treatment information from two out of four study sites may have biased our findings. In the two excluded sites, where newly diagnosed MDR-TB patients were treated in centers other than where the Xpert instrument was placed, it is important to repeat analysis after improvements are made to the recording system. This can be done by strengthening data exchange with district TB centers, pre-trial detention centers, the penitentiary system, and HIV centers.

A third limitation was that Xpert and phenotypic DST were performed in parallel on the same individuals. As a result, it was not possible to compare both tests beyond diagnosis, meaning that we could not assess time to treatment, proportion of patient that started treatment, etc. This would require a study design with two different cohorts, for example a before-after observational cohort or randomized controlled trial.

Following the successful roll-out of the initial four USAID-funded Xpert instruments, the technique is being scaled up in Kazakhstan and involves placing a large number of locally-funded machines at additional provincial laboratories as well as district laboratories. Placing instruments at more peripheral levels increases access to a rapid diagnosis and has the added benefit of reducing time and costs of sample referral. A possible pitfall is that a machine at district level will cover a smaller population than a machine at provincial level and may therefore be underutilized. This pilot showed that the first four sites in Kazakhstan performed on average 159 Xpert tests per month per site, which is 66% of the realistic maximal instrument throughput of 240 tests per month if a 4-module Xpert machine is expected to run 12 tests per day.[15] This workload is high compared to other countries where Xpert was introduced under similar programmatic conditions,[16] but could still be increased by 34%. Based on these estimations, the NTP planned to procure Xpert machines with two instead of four functional test modules at a reduced price of 12,280 USD per machine for sites with lower expected workload. Further, this pilot found high proportions of RR-TB among (former) prisoners and PLWHA suspected of TB, which was in line with a recent study showing largely overlapping risk factors for HIV and MDR-TB among TB patients in Kazakhstan.[17, 18] Increasing referrals from penitentiary and HIV centers to TB centers could maximize the yield of Xpert with regard to RR-TB case detection and treatment. Another option would be to expand Xpert testing to all presumptive TB cases regardless of their risk group, which is justifiable given the high prevalence of MDR-TB among new TB patients. Other issues to consider when placing machines at peripheral level are challenges for central laboratories (NRL and regional) to manage distribution of supplies and supervision of operation, especially in a country like Kazakhstan where weather conditions often impede access to remote sites. Considerations for future placement and eligible groups for Xpert testing are being assessed in an ongoing cost-effectiveness analysis.

## Conclusions

Xpert was successfully introduced in the NRL and three provincial laboratories in Kazakhstan. Compared to conventional diagnosis with culture and DST, Xpert had a higher percentage of failed tests, shorter result turn-around-time and excellent concordance to detect RR-TB. Uptake of Xpert by laboratory staff and clinicians was rapid, based on the high throughput of tests from the start of installation and the short time needed to start patients on first- and second-line TB treatment after diagnosis. When Xpert is scaled up in the country to district level laboratories, the NTP could consider testing all presumptive TB patients with Xpert regardless of their risk group in order to avoid underutilization of the instruments. Primary efforts should to strengthening referral mechanisms between prisons and TB centers, and HIV clinics and TB centers, in order to increase rapid detection of RR-TB using Xpert among underserved risk groups.
