# Correction: Effect of Introducing Xpert MTB/RIF to Test and Treat Individuals at Risk of Multidrug-Resistant Tuberculosis in Kazakhstan: A Prospective Cohort Study

**DOI:** 10.1371/journal.pone.0136368

**Published:** 2015-08-19

**Authors:** Sanne Christine van Kampen, Aigul Tursynbayeva, Aliya Koptleuova, Zauresh Murzakhmetova, Lyazzat Bigalieva, Moldir Aubakirova, Svetlana Pak, Susan van den Hof

The fourth author's name is spelled incorrectly. The correct name is: Zauresh Murzakhmetova. The correct citation is: van Kampen SC, Tursynbayeva A, Koptleuova A, Murzakhmetova Z, Bigalieva L, Aubakirova M, et al. (2015) Effect of Introducing Xpert MTB/RIF to Test and Treat Individuals at Risk of Multidrug-Resistant Tuberculosis in Kazakhstan: A Prospective Cohort Study. PLoS ONE 10(7): e0132514. doi:10.1371/journal.pone.0132514.
